# Orexin/Hypocretin-1 Receptor Antagonism Selectively Reduces Cue-Induced Feeding in Sated Rats and Recruits Medial Prefrontal Cortex and Thalamus

**DOI:** 10.1038/srep16143

**Published:** 2015-11-05

**Authors:** Sindy Cole, Heather S. Mayer, Gorica D. Petrovich

**Affiliations:** 1 Department of Psychology, Boston College, 140 Commonwealth Avenue Chestnut Hill, MA 02467-3807 United States

## Abstract

The orexin/hypocretin system is important for reward-seeking behaviors, however less is known about its function in non-homeostatic feeding. Environmental influences, particularly cues for food can stimulate feeding in the absence of hunger and lead to maladaptive overeating behavior. The key components of the neural network that mediates this cue-induced overeating in sated rats include lateral hypothalamus, amygdala, and medial prefrontal cortex (mPFC), yet the neuropharmacological mechanisms within this network remain unknown. The current study investigated a causal role for orexin in cue-driven feeding, and examined the neural substrates through which orexin mediates this effect. Systemic administration of the orexin-1 receptor (OX1R) antagonist SB-334867 had no effect on baseline eating, but significantly reduced cue-driven consumption in sated rats. Complementary neural analysis revealed that decreased cue-induced feeding under SB-334867 increased Fos expression in mPFC and paraventricular thalamus. These results demonstrate that OX1R signaling critically regulates cue-induced feeding, and suggest orexin is acting through prefrontal cortical and thalamic sites to drive eating in the absence of hunger. These findings inform our understanding of how food-associated cues override signals from the body to promote overeating, and indicate OX1R antagonism as a potential pharmacologic target for treatment of disordered eating in humans.

Environmental and social factors critically control our appetites and food consumption. In particular, food-associated environmental cues have been shown to powerfully stimulate eating in sated (not food-deprived) children and adults^1^,^2^. Recent research has employed an animal model of this cue-induced feeding to delineate the critical neural circuits^3^. In this model, through repeated pairings, rats learn to associate a neutral stimulus (e.g., a tone) with delivery of a palatable food, such that the cue becomes a signal for the food. The ability of the cue to stimulate overeating is later assessed in sated rats by presenting the tone and measuring food consumption. In the presence of the cue, rats eat significantly greater amounts of food.

The neural circuitry underlying this non-homeostatic eating includes the basolateral and basomedial amygdalar nuclei (BLA and BMA), the lateral hypothalamic area (LHA), and the medial prefrontal cortex (mPFC)^4^,^5^. Additionally, specific pathways from the BLA/BMA and mPFC to the LHA are strongly activated by the food cue^6^, further implicating this network. In contrast, very little is known about the neuropharmacological mechanisms that act within this circuit to promote overeating. One promising candidate is the neuropeptide orexin/hypocretin.

Orexin is synthesized exclusively by neurons in the lateral hypothalamus^7^,^8^, a region traditionally associated with the initiation of feeding. This confined population of neurons projects extensively throughout the brain^9^, and these widespread connections allow orexin to modulate numerous eating and reward-seeking behaviors. Central administration of orexin increases consumption in rats^8^,^10^, implicating orexin in homeostatic regulation of feeding. However, presentation of food- or reward-predictive cues alone is sufficient to strongly activate orexin neurons^11^,^12^,^13^, suggesting a broader role for orexin in mediating reward-driven motivated behaviors. Consistent with this view, orexin receptor blockade significantly reduces self-administration for palatable foods in a variety of preparations. For example, orexin receptor antagonism decreased self-administration of high-fat pellets^14^, sucrose^15^, and saccharin^16^. Systemic orexin blockade has also been shown to reduce motivation to respond for high-fat pellets^17^, and to decrease cue-induced reinstatement of sucrose- and saccharin-seeking behavior^15^,^16^.

Orexin mediates its effects via two G protein-coupled receptors, OX1R and OX2R, which are differentially distributed throughout the brain^18^,^19^. Accordingly, there is heterogeneity in the function of these receptors, with OX1R activation more closely associated with appetitive and reward behaviors, while OX2R has been predominantly associated with sleep/wake transitions and arousal^20^,^21^,^22^. Selective antagonism of OX1R has been shown to reduce both homeostatic (nocturnal and post-fast) feeding and orexin-induced intake^23^, while blockade or knockdown of OX1R reduced high-fat diet consumption^11^,^24^. Similarly, OX1R antagonism has attenuated operant responding for food pellets^25^ and palatable, high-fat pellets^14^, and also prevented cue- and stress-induced reinstatement of sucrose-seeking^15^,^26^.

Importantly, orexin fibers and receptors are strongly expressed within the cue-induced feeding circuitry (mPFC, BLA, BMA)^18^,^19^,^27^, suggesting a role for orexin in mediating this overeating effect. In accordance with this hypothesis, we previously showed that presentation of a tone food-cue activates (Fos induction) orexin neurons^13^. Therefore, here we used systemic administration of an OX1R antagonist coupled with Fos immunohistochemistry to first determine whether OX1R signaling is critical for cue-induced feeding, and then to elucidate the neuroanatomical location/s of its action. Specifically, we examined the key components of the cue-induced feeding circuit (mPFC, BLA, BMA, LHA) as well as two connected regions (the paraventricular thalamic nucleus [PVT] and the central amygdalar nucleus [CEA]) where OX1R action mediates hedonic eating and taste learning respectively^24^,^28^.

## Results

### Experiment 1: Effect of SB-334867 on cue-induced feeding in sated rats

In Experiment 1 we determined the effect of systemic injections of the OX1R antagonist SB-334867 on cue-induced feeding. During training, rats received either tone-food pairings (Paired, n = 7), or tone alone presentations followed by food pellet delivery later in the home cage (Unpaired, n = 7). All animals were then tested for consumption of food pellets under SB-334867 and under vehicle, in a counterbalanced order across two consecutive days. Each test consisted of a baseline consumption test (stimulus-free), followed by a cue consumption test (tone presentations).

#### Body Weight

Rats in the Paired and Unpaired groups had similar body weights at the beginning of training (P: 297 ± 3 g, U: 291 ± 5 g), and prior to testing (P: 297 ± 5 g, U: 287 ± 6 g). Two-way independent samples t-Tests confirmed there were no significant differences in body weight between groups at either time point (t_(12)_ values = 1.05, 1.27 respectively, *p* values > 0.05).

#### Training Chow Consumption

There were no differences between groups in the overall amounts of chow consumed daily or across training days. A group (Paired, Unpaired) by training day ANOVA on daily chow consumption yielded a significant linear trend of training day (*F*_(1,12)_ = 79.90, P < 0.0001), confirming that chow consumption increased across training days. There was no significant effect of group (*F*_*(1,12)*_ = 2.65, *p* > 0.05), and no group by training day interaction (*F*_*(1,12)*_ < 1, *p* > 0.05).

### Test

#### Baseline Test

**Figure 1:**
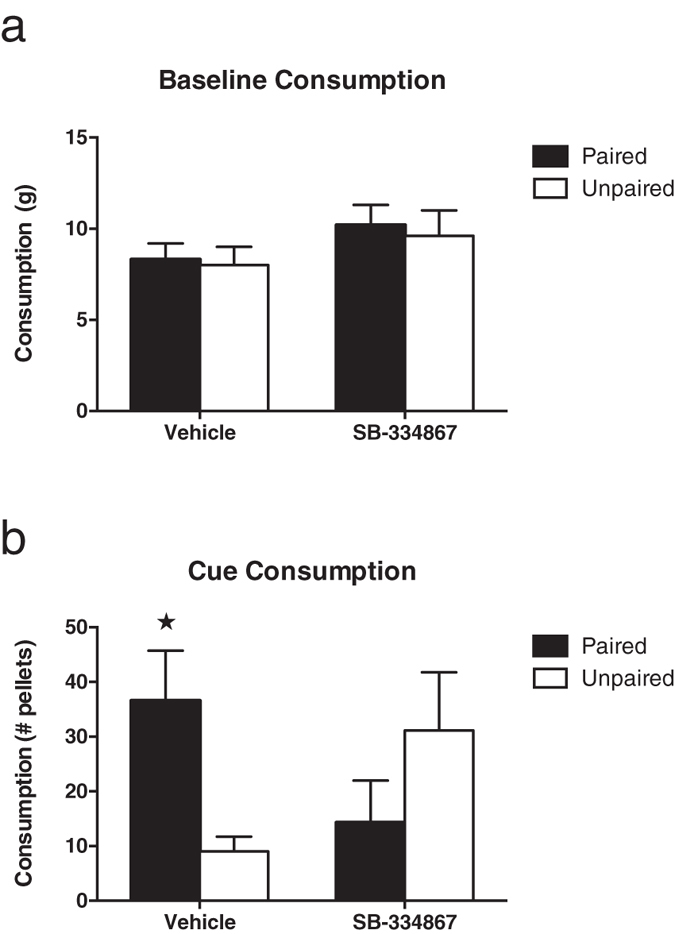
Consumption of food pellets (mean ± SEM) during (**a**) the stimulus-free baseline test and (**b**) test with cue-food presentations. Asterisk indicates a significant difference between Paired and Unpaired groups *p* < 0.05.

Food pellet consumption during the stimulus-free baseline tests are shown in Fig. 1a. Rats in the Paired and Unpaired groups ate similar amounts of the pellets during the baseline test, regardless of whether they had received vehicle or SB-334867. A group by drug treatment (vehicle, SB-334867) ANOVA showed no significant effect of group (*F*_*(1,12)*_ < 1, *p* > 0.05), or drug treatment (*F*_*(1,12)*_ = 2.80, *p* > 0.05), and no significant group by drug treatment interaction (*F*_*(1,12)*_ < 1, *p* > 0.05).

#### Cue Test

Figure 1b shows the number of food pellets consumed during the cue tests. Under vehicle the Paired group showed cue-induced feeding, but SB-334867 treatment attenuated this effect. A group by drug treatment ANOVA yielded a significant interaction (*F*_(1,12)_ = 12.25, *p* = 0.004). Follow-up analyses showed that under vehicle the Paired group ate significantly more than the Unpaired group (*F*_(1,12)_ = 9.77, *p* = 0.009), confirming cue-induced feeding. However, following SB-334867 treatment animals in the Paired group no longer showed this effect, with no significant difference in consumption between groups (*F*_*(1,12)*_ = 1.90, *p* > 0.05).

### Experiment 2: Fos induction during cue-induced feeding under SB-334867

In Experiment 2 we determined whether blockade of cue-induced feeding after systemic injection of SB-334867 alters activity patterns in brain regions of interest. All rats received tone-food pairings throughout training. On test day, all rats received a drug-free baseline test, followed by an injection of either SB-334867 or vehicle. They then received either a cue test (VEH Cue: n = 6, SB Cue: n = 6), or remained in their home cage with no food to control for non-specific effects of the drug on neural activity (VEH Home: n = 6, SB Home: n = 6). Brain tissue was then collected for later processing for Fos induction as a marker of neuronal activation.

#### Body Weight

All rats had similar body weights at the beginning of training (SB Cue: 327 ± 7 g, VEH Cue: 329 ± 5 g, SB Home: 343 ± 7 g, VEH Home: 334 ± 7 g), and prior to testing (SB Cue: 326 ± 8 g, VEH Cue: 327 ± 5 g, SB Home: 336 ± 2 g, VEH Home: 330 ± 10 g). One-way ANOVAs confirmed there were no significant differences in body weight between groups at either time point (*F*_(3,23)_ values = 1.63, 0.45 respectively, *p* values > 0.05).

#### Training Chow Consumption

All groups consumed similar overall amounts of chow across training. There was a significant linear trend of training day (*F*_(1,20)_ = 206.61, *p* < 0.0001), confirming that chow consumption increased across training days. There were no significant group differences or interactions (*F*_*(1,20)*_ values < 1.5, *p* values > 0.05).

### Test

#### Baseline Test

**Figure 2:**
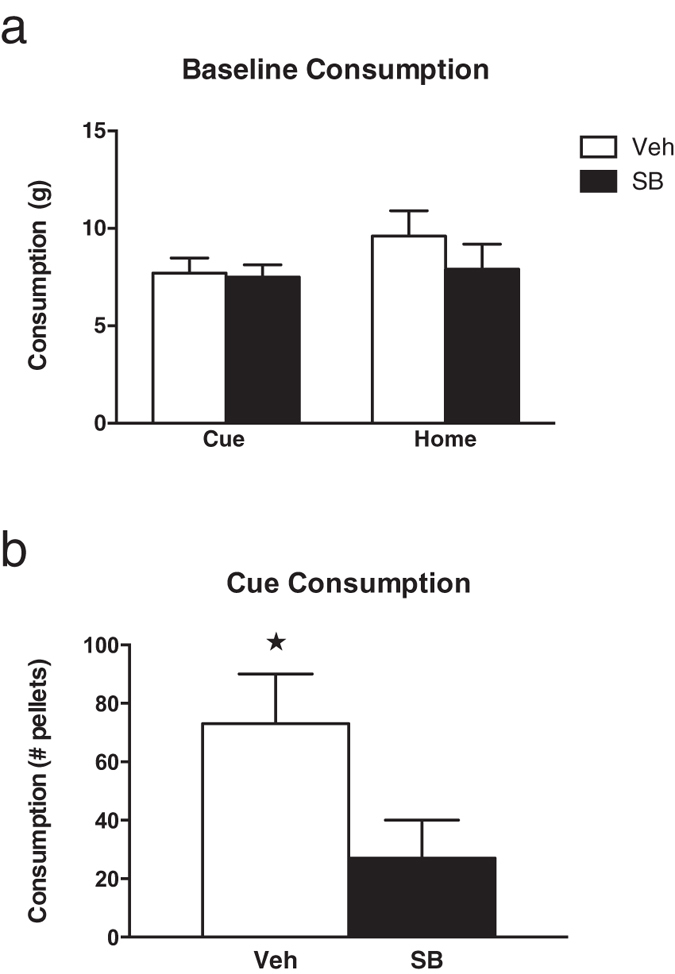
Consumption of food pellets (mean ± SEM) during (**a**) the stimulus-free baseline test and (**b**) test with cue-food presentations. Asterisk indicates a significant difference compared to the SB Cue group *p* < 0.05.

Consumption during the drug-free baseline test is shown in Fig. 2a. All rats ate similar amounts of pellets. A one-way ANOVA revealed no significant group differences (*F*_*(3,23)*_ < 1, *p* > 0.05).

#### Post-injection Chow Consumption

During the 30 minutes immediately following injection all rats ate similarly small amounts of chow in their home cage (all <0.2 g). A group (Cue, Home) by drug treatment (vehicle, SB-334867) ANOVA revealed no significant effect of group (*F*_*(1,20)*_ = 3.98, *p* > 0.05), or drug treatment (*F*_*(1,20)*_ < 1, *p* > 0.05), and no significant group by drug treatment interaction (*F*_*(1,20)*_ < 1, *p* > 0.05).

#### Cue Test

Figure 2b shows the number of pellets consumed during the cue test (tone presentations) for animals in the two Cue groups. Rats that received SB-334867 treatment before test ate significantly less food than those that received vehicle treatment. A two-way independent samples t-Test confirmed this reduction in cue-induced feeding by SB-334867 (t_(10)_ = 2.31, *p* = 0.043).

### Fos Induction

Fos-positive neurons were observed in all brain regions examined (Table 1). Data from the following were not collected due to tissue damage: one SB Cue (ACAd, PL, ILA, BMAp), and two VEH Home (BLAp, BMAp).

**Figure 3:**
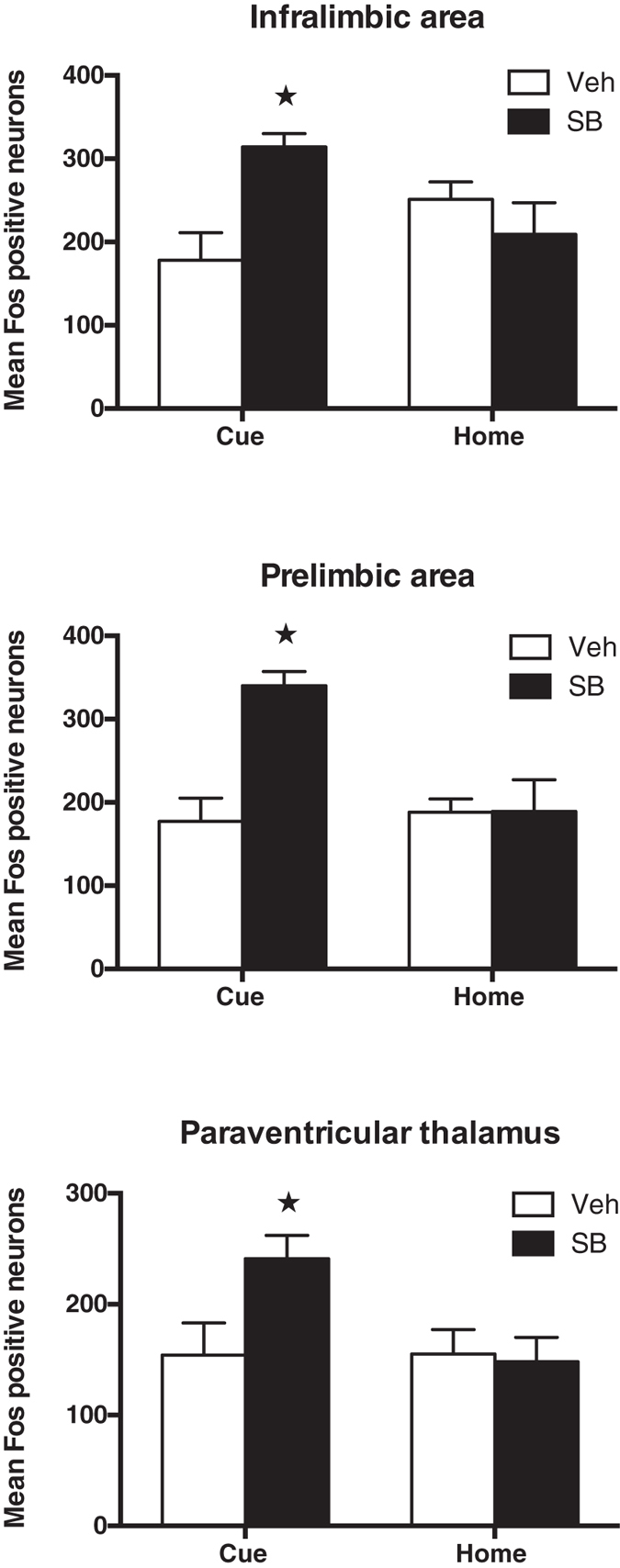
Total number of Fos-positive neurons in the infralimbic area, the prelimbic area, and the anterior part of the paraventricular thalamic nucleus (mean ± SEM). Asterisk indicates a significant difference compared to the VEH Cue group *p* < 0.05.

**Figure 4:**
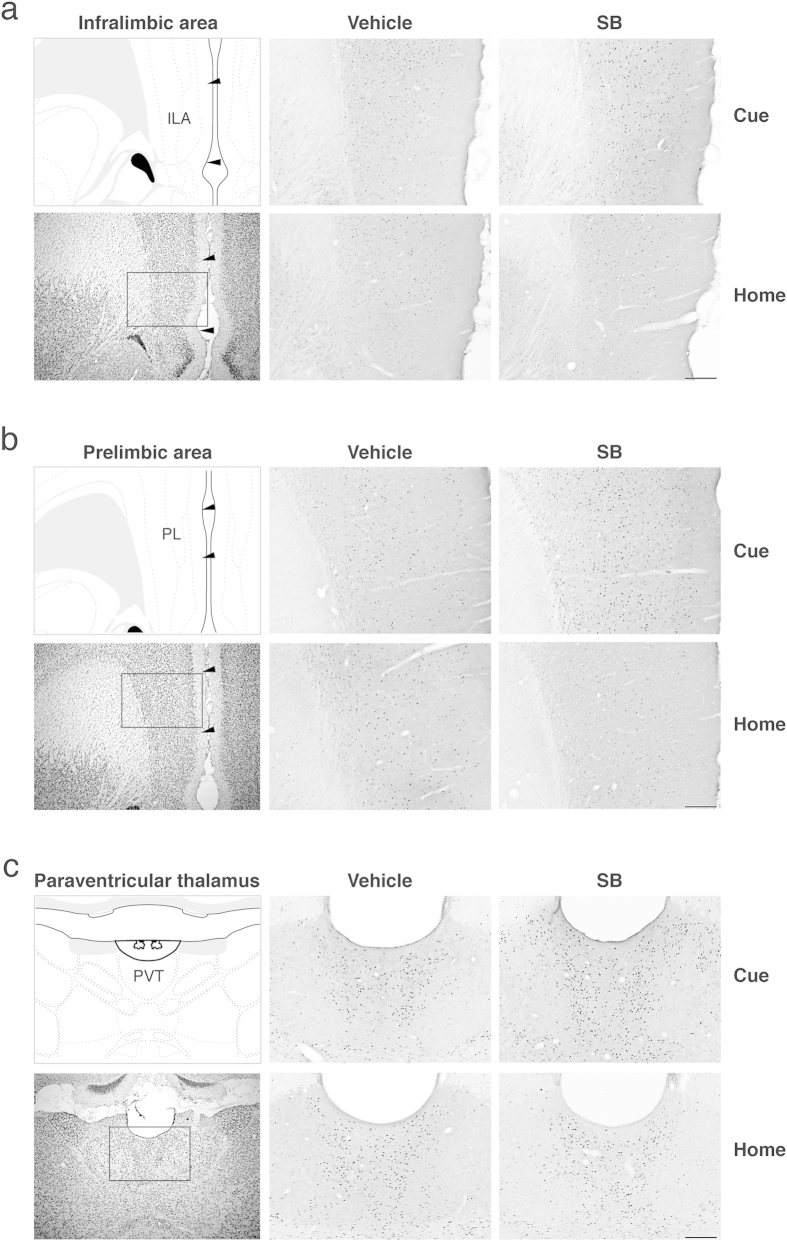
Representative photomicrographs of Fos induction during the cue test (Cue) or equivalent time period spent in the home cage (Home) in (**a**) the infralimbic area (ILA), (**b**) the prelimbic area (PL), and (**c**) the anterior part of the paraventricular thalamic nucleus (PVT). For each region cytoarchitectonic borders are depicted on a modified atlas template^49^ and on a corresponding thionin-stained section (left top and bottom panels), and each box depicts the area shown in the corresponding Fos images. Rats were trained with tone-food pairings, and then sated before testing. Following systemic injection of either an OX1R antagonist (SB) or vehicle (VEH), rats were either tested for consumption during tone-food presentations (Cue) or remained in their home cage with no stimuli (Home). Images were captured at 10 x using an Olympus DP72 camera and DP2-BSW software (Olympus America Inc, Center Valley, PA, USA) and processed for contrast, sharpening, and brightness with Adobe Photoshop CS5 (Adobe Systems Incorporated, San Jose, CA, USA). Scale bar = 200 μm.

We observed a significant increase in Fos induction that was specific to the SB Cue group in three regions. This was evident as a significant group (Cue, Home) by drug treatment (vehicle, SB-334867) interaction in the ILA (*F*_(1,19)_ = 10.91, *p* = 0.004), the PL (*F*_(1,19)_ = 10.64, *p* = 0.004), and the PVTa (*F*_(1,20)_ = 4.74, *p* = 0.042). Further analyses demonstrated that in each of these regions the SB Cue group had significantly greater Fos induction than the VEH Cue group (*F* values = 12.15, 20.73, 8.18, *p* values = 0.002, <0.0001, =0.010 respectively), but there were no such differences between the Home control groups (*F* values < 1.3, *p* values > 0.05). Figures 3 and 4 show the average total counts and representative photomicrographs from these three regions.

Exposure to the cue test increased Fos expression in the ACAd and BMAp, independent of drug treatment. In both of these regions a group (Cue, Home) by drug treatment (vehicle, SB-334867) ANOVA found a significant main effect of group (*F* values = 11.37, 10.24, *p* values = 0.003, 0.005 respectively), indicating higher levels of Fos induction in the Cue groups compared to the Home groups. There was no significant effect of drug treatment, or group by drug treatment interaction (*F* values < 4.2, *p* values > 0.05). The only other region to show this pattern of Fos induction was the BLAp, where the main effect of group approached significance (*p* = 0.07). There were no significant group differences detected in any of the other brain regions examined.

## Discussion

Here, we showed that the OX1R antagonist SB-334867 decreased food intake driven by a food cue selectively, with no effect on baseline eating. In addition, we found elevated Fos induction within the mPFC (PL and ILA) and PVTa in rats tested for cue-induced feeding under SB-334867, compared to rats tested under vehicle. These results demonstrate that orexin signaling at OX1Rs is necessary for cue-induced feeding and that these prefrontal cortical and thalamic regions are important sites of its action.

These effects were specific to a decrease in motivation to eat, and not due to a non-selective effect of the drug on general arousal or locomotor activity. Orexin antagonists, including SB-334867, have been shown to produce sedative effects in rodents^29^. Nevertheless, the reduction in food intake we observed cannot be attributed to such properties of the drug. SB-334867 had no effect during the baseline test (Experiment 1), demonstrating that it did not affect the animals’ ability to locomote towards the food cup, or to retrieve or consume the food. The dose used (20 mg/kg) was chosen because it has significantly modulated reward motivated behaviors without affecting locomotor activity (e.g.,^26^), and is lower than the dose that produced motoric effects^29^. Therefore, the current data show that SB-334867 specifically reduced non-homeostatic feeding during the cue test, in agreement with the proposed orexin function to drive behavior under increased motivational states, translating motivation into action^30^,^31^,^32^.

We found increased Fos induction in the PL, ILA, and PVTa when cue-induced feeding was blocked by SB-334867. This effect was specific to reduction of feeding, and not simply due to a non-specific effect of the drug, because there was no similar increased Fos induction in home cage controls given SB-334867. Therefore, this change in activity reveals orexin action within mPFC and PVTa during cue-induced feeding.

This is the first demonstration that orexin signaling at OX1Rs in the mPFC may be important in mediating feeding behavior. Nevertheless, these findings complement other recent studies implicating PL and ILA in the control of feeding and reward-driven behaviors (e.g.,^5^,^33^,^34^,^35^). There was also a significant change in the PVTa, a region interconnected with key components of the cue-induced feeding network (PL, ILA, BLA/BMA, LHA)^36^,^37^. The PVT is important in negative motivation and affect as well as arousal, but it also mediates feeding and reward behaviors and associated motivation (see^38^,^39^). More specifically, a recent study demonstrated knockdown of OX1Rs in the PVT reduced over-consumption of a palatable food in sated rats^24^. Together, these results suggest that orexin is specifically acting within this network to drive non-homeostatic feeding. Further studies are needed to confirm whether PVTa is necessary for cue-induced feeding, and to determine the exact contribution of orexin signaling within these prefrontal and thalamic regions.

We did not observe significant differences in LHA Fos induction, which suggests that orexin is not acting locally during cue-induced feeding, in agreement with higher expression of OX2Rs than OX1Rs^18^. However, the LHA is a heterogeneous structure, and it is possible that a small subset of neurons mediates cue-induced feeding (e.g., orexin-containing neurons). Further work is needed to investigate this possibility.

Interestingly, here we found that systemic OX1R antagonism increased Fos expression in a restricted system of PL, ILA and PVTa neurons. This strongly suggests that SB-334867 removed inhibition within these areas, and in turn implies that during cue-induced feeding orexin signaling through OX1R inhibits these targets. This release from inhibition by SB-334867 could be achieved by either direct removal of inhibition (orexin action is inhibitory), or through disinhibition (orexin action is excitatory, but targeting GABAergic cells). The latter mechanism has been observed in recent studies and is therefore more likely. For example, Aracri and colleagues demonstrated that in the PFC, application of orexin excited fast-spiking interneurons, causing the release of GABA onto pyramidal cells^40^. Furthermore, this effect was specifically mediated by OX1Rs (also see^41^,^42^). These findings suggest that the increased Fos in the mPFC is the result of disinhibition.

Given that there are no inhibitory neurons in the rodent midline thalamus^43^, increased activation in PVT could arise from multiple sources, including disinhibited layer 5/6 mPFC pyramidal cells, other activated inputs (e.g., amygdala, LHA), or from local action of presynaptic OX1Rs. Furthermore, given that mPFC and PVTa are reciprocally connected^44^,^45^, these two regions could form a functional loop that would further enhance orexin action within this circuit.

Interestingly, even though SB-334867 treatment reduced cue-driven consumption, there was no corresponding suppression of Fos induction in any of the brain regions examined. This suggests that blockade of OX1R function did not prevent the activation of any of the critical neural sites investigated here. It is possible however, that additional structures important for aspects of cue-driven overeating were inhibited. Candidate areas include the nucleus accumbens (ACB), and the ventral pallidum (VP) (substantia innominata), where modulation of the orexin system alters feeding behavior and hedonic reactivity to food. Infusion of Orexin A into the ACB increased food consumption^46^,^47^, blockade of ACB opioid receptors prevented Orexin A-induced feeding^48^, and Orexin A infusion into the VP significantly increased positive ‘liking’ orofacial reactions to sucrose^49^. However, whether orexin signaling in the ACB and/or VP is critical for non-homeostatic eating driven by environmental learned cues requires further examination.

In summary, this study demonstrated that pharmacological OX1R blockade specifically decreased food intake driven by a learned food cue. This blockade of cue-induced feeding by OX1R antagonism also increased Fos induction in the mPFC (PL and ILA) and PVTa. These findings clearly show orexin signaling at OX1Rs is critical in cue-induced feeding, and suggest that orexin is acting through these cortical and thalamic sites to drive eating in the absence of hunger.

## Methods

### Animals

Experimentally naïve, male Long-Evans rats (275–300 g) from Charles River Laboratories (Portage, MI, USA) were used. The animals were individually housed with *ad libitum* access to food and water except when otherwise noted. The colony room was maintained at 21 °C on a 12 hour light/dark cycle (lights on 06:00) and all behavioral testing was conducted during the light phase of the cycle. The housing and testing procedures were in accordance with the National Institute of Health *Guidelines for Care and Use of Laboratory Animals* and approved by the Boston College Institutional Animal Care and Use Committee.

### Apparatus

Training and testing were conducted in a set of behavioral chambers described previously^50^. The floor in each chamber was covered with a black Plexiglas insert. Training stimuli were a 150s tone (75 dB, 2 kHz), and 50 food pellets (5TUL, 45 mg; Test Diets, Richmond, IN, USA; 3.4 kcal/g; 20% protein, 13% fat, 67% carbohydrate) delivered to the food-cup of each chamber unless otherwise noted. The stimuli were controlled by the GraphicState 3.0 software system (Coulbourn Instruments, Allentown, PA, USA).

### Drugs

The OX1R antagonist SB-334867 (Tocris Bioscience, Ellisville, MO, USA), was dissolved in 10% (2-Hydroxypropyl)-β-cyclodextrin (Sigma-Aldrich, St. Louis, MO, USA) and 2% dimethyl sulfoxide (Sigma-Aldrich) in sterile water, and administered (20 mg/kg) via intraperitoneal (i.p.) injection at a volume of 2 ml/kg.

### Experiment 1: Effect of an OX1R antagonist on cue-induced feeding in sated rats

Animals were assigned to experimental group conditions, which were matched for baseline *ad libitum* chow consumption (measured twice for 24 hours) and body weight. A day prior to the start of training, chow was removed and all animals received 1 g of the food pellets in their home cage to familiarize them with the pellets. Animals remained food-restricted throughout training, with access to chow for one hour each day immediately following the completion of each training session.

#### Training

There were eight, 30-minute behavioral training sessions. During each session, rats in the Paired condition received two presentations of a tone. Each tone presentation overlapped and co-terminated with delivery of 50 food pellets. Rats in the Unpaired condition also received two presentations of the tone that overlapped with the sound of the food pump, but no food was delivered. 30–120 minutes following each session, the Unpaired group received food pellets in their home cage, with the timing and quantity matched to that given to the Paired group. Upon completion of training all rats were returned to *ad libitum* chow consumption for 3 days before testing commenced.

#### Test

All animals were tested across two consecutive days, under the OX1R antagonist SB-334867 and under vehicle, in a counterbalanced order. On each day animals received the assigned injection and then remained in home cages for 30 minutes. Testing then consisted of a baseline consumption test, followed by the cue test.

#### Baseline Test

*A*nimals were placed into the behavioral chamber for 20 minutes with *ad libitum* access to 7 g of food pellets. The rats were briefly removed from the chamber while any remaining pellets were collected and weighed, and then placed back into the chamber for an additional 20 minutes with another 7 g of pellets. Again any remaining pellets were collected and weighed.

#### Cue Test

Immediately following completion of the baseline test, rats were placed back into the behavioral chamber for a cue test session (approximately 10 minutes). In this session they received three tones, each of which co-terminated with delivery of 50 food pellets. All rats were then returned to the colony room and any remaining food pellets were counted.

### Experiment 2: Fos induction during cue-induced feeding under SB-334867

#### Training

All animals received training as described for the Paired condition in Experiment 1. Upon completion of training all rats were returned to *ad libitum* chow consumption for 3–4 days before testing commenced.

### Test

#### Baseline Test

All animals underwent drug-free baseline testing as described in Experiment 1, except each exposure was 15 minutes.

#### Cue Test

Immediately following the baseline test animals were placed back into home cages, returned to the colony room, and injected with either SB-334867 or vehicle. 30 minutes later half of the animals were placed into the behavioral chamber for the cue test as described in Experiment 1, following which they were returned to the colony room in home cages with no access to food (SB Cue, VEH Cue). The other half of the animals remained in home cages to control for non-specific effects of the drug on neural activity (SB Home, VEH Home). For these animals chow was removed 30 minutes following injections to match the timing of chow removal for the Cue animals, and to prevent any Fos induction due to food consumption. Animals were perfused, and the brains removed, two hours from the beginning of the cue test or the equivalent time for Home controls.

### Histological Procedures

Rats were anaesthetized by an i.p. injection of tribromoethanol (375 mg/kg body weight) and transcardially perfused with 0.9% saline followed by ice-cold 4% paraformaldehyde in 0.1 M borate buffer (pH 9.4). The brains were stored for 20–24 hours at 4 °C in the fixative with 12% sucrose and then rapidly frozen in hexanes (Thermo Fisher Scientific, Pittsburgh, PA, USA) cooled with dry ice and stored at −80 °C. Frozen brains were cut into 30 μm coronal slices using a sliding microtome (Leica SM200R) and collected into four, serially adjacent, sets. One series of sections was used to identify Fos with standard immunohistochemistry. Free-floating sections were rinsed with potassium phosphate-buffered saline (KPBS), and then incubated with anti-*c-fos* antibody raised in rabbit (1:40 000, Ab-5, PC38; Calbiochem, San Diego, CA, USA) in a blocking solution of KPBS containing 2% normal goat serum (NGS) (S-1000; Vector Laboratories, Burlingame, CA, USA) and 0.3% Triton X-100 (Sigma–Aldrich) for 72 hours at 4 °C with gentle agitation. Sections were then brought to room temperature, rinsed with KPBS, incubated with biotinylated secondary antibody against rabbit (1:200, BA-1000; Vector Laboratories) in the blocking solution for 90 minutes, rinsed in KPBS, incubated in avidin biotin complex (ABC, PK-6100; Vector Laboratories) for 90 minutes and again rinsed in KPBS. Neurons immunoreactive for Fos were visualized as brown after a one-minute incubation in 3, 3’-diaminobenzidine (SK-4100; Vector Laboratories). Sections were then rinsed, mounted on SuperFrost slides (Thermo Fisher Scientific), dried at 40 °C, dehydrated through graded alcohols, cleared in xylenes, and coverslipped with DPX Mountant (Electron Microscopy Services, Hatfield, PA, USA). A second series of sections were mounted from KPBS onto chrome alum/gelatin-coated slides and stained with thionin for identification of cytoarchitectonic borders.

### Image Acquisition and Analysis

The analysis followed parcellation and nomenclature as defined in the Swanson atlas (2004), except for the LHA and the PVT, and these exceptions are described below. Regions of interest were selected based on their involvement in non-homeostatic feeding and appetitive learning, and expression of OX1R. Image acquisition and neuronal counting followed procedures described previously^50^. Images were acquired bilaterally for each cell group analyzed and left and right counts were summed for each rat, then averaged for each group, resulting in a mean total of Fos-labeled neurons.

The following regions of interest were analyzed. Levels refer to the Swanson^51^ atlas level, and measurements refer to mm from bregma. Within the mPFC (Level 9, +2.80 mm) we analyzed the anterior cingulate area, dorsal part (ACAd), the prelimbic area (PL), and the infralimbic area (ILA). Within the amygdala, we analyzed the anterior (BLAa) (Level 27, −2.00 mm) and posterior (BLAp) (Level 30, −3.25 mm) parts of the BLA, the posterior part of the BMA (BMAp) (Level 30, −3.25 mm), and the medial (CEAm), lateral (CEAl), and capsular (CEAc) parts of the CEA (Level 27, −2.00 mm). For the LHA (Level 29, −2.85 mm) we defined two areas for analysis: the perifornical area (LHApf) that contained the juxtadorsomedial and suprafornical nuclei^52^,^53^, and the lateral area (LHAl), which included the region lateral to the LHApf, and was bordered dorsally by a line that matches the dorsal end of the third ventricle and ventrally by the ventral edge of the fornix. Within the PVT, we sampled from its anterior (PVTa) (Level 26, −1.78 mm) and posterior (PVTp) (Level 30, −3.25 mm) areas, separately.

### Statistical Analysis

Body weight and food consumption data were analyzed using either independent sample t-Tests, or ANOVAs, as appropriate. Counts of Fos-positive neurons were analyzed for each structure using a two-way ANOVA with group (Cue, Home) and drug treatment (SB-334867, Vehicle) as between-subjects factors. In Experiment 1 two rats exhibited unusual baseline consumption at test (beyond 2 SEM), in Experiment 2 two rats failed to consistently consume all of the food pellets during training, and six exhibited unusual baseline eating at test (beyond 2 SEM); these rats were excluded from behavioral and neuronal analyses. The number of rats reported refers to those included in the statistical analyses. The statistical packages PSY^54^ and SPSS (v.21) were used and type I error rate was controlled at 0.05.

## Additional Information

**How to cite this article**: Cole, S. *et al.* Orexin/Hypocretin-1 Receptor Antagonism Selectively Reduces Cue-Induced Feeding in Sated Rats and Recruits Medial Prefrontal Cortex and Thalamus. *Sci. Rep.* **5**, 16143; doi: 10.1038/srep16143 (2015).

## Figures and Tables

**Table 1:** Number of Fos-Positive Neurons for All Brain Regions Examined.

Brain Region	Cue		Home	
	Vehicle	SB-334867	Vehicle	SB-334867
ACAd^2^	217 (46)	338 (30)	155 (26)	172 (40)
PL^1^	177 (28)	340 (17)	188 (16)	189 (38)
ILA^1^	178 (33)	314 (16)	251 (21)	209 (38)
BLAa	71 (15)	79 (21)	82 (21)	61 (10)
BLAp^3^	74 (14)	76 (11)	45 (13)	58 (14)
BMAp^2^	93 (8)	103 (21)	47 (14)	60 (27)
CEAl	46 (9)	54 (14)	33 (11)	40 (9)
CEAm	79 (11)	78 (6)	85 (18)	80 (12)
CEAc	59 (3)	68 (9)	53 (7)	57 (11)
PVTa^1^	154 (29)	241 (21)	155 (22)	148 (22)
PVTp	118 (18)	119 (15)	109 (12)	132 (12)
LHApf	149 (23)	176 (9)	161 (23)	141 (26)
LHAl	66 (16)	61 (5)	60 (14)	44 (10)

Fos counts are displayed as a mean total number for each area (SEM).

^1^Group by drug treatment interaction *p* < 0.01.

^2^Main effect of group (cue vs. home) *p* < 0.01.

^3^Main effect of group (cue vs. home) *p* = 0.07.
